# Reduced drug incorporation into DNA and antiapoptosis as the crucial mechanisms of resistance in a novel nelarabine-resistant cell line

**DOI:** 10.1186/1471-2407-14-547

**Published:** 2014-07-29

**Authors:** Takahiro Yamauchi, Kanako Uzui, Rie Nishi, Hiroko Shigemi, Takanori Ueda

**Affiliations:** Department of Hematology and Oncology, Faculty of Medical Sciences, University of Fukui, 23-3, Shimoaizuki, Matsuoka, Fukui 910-1193 Japan

**Keywords:** Ara-G, Ara-GTP, Nelarabine, Resistance, T-ALL

## Abstract

**Background:**

Nine-beta-D-arabinofuranosylguanine (ara-G), an active metabolite of nelarabine, enters leukemic cells through human Equilibrative Nucleoside Transporter 1, and is then phosphorylated to an intracellular active metabolite ara-G triphosphate (ara-GTP) by both cytosolic deoxycytidine kinase and mitochondrial deoxyguanosine kinase. Ara-GTP is subsequently incorporated into DNA, thereby inhibiting DNA synthesis.

**Methods:**

In the present study, we developed a novel ara-G-resistant variant (CEM/ara-G) of human T-lymphoblastic leukemia cell line CCRF-CEM, and elucidated its mechanism of ara-G resistance. The cytotoxicity was measured by using the growth inhibition assay and the induction of apoptosis. Intracellular triphosphate concentrations were quantitated by using HPLC. DNA synthesis was evaluated by the incorporation of tritiated thymidine into DNA. Protein expression levels were determined by using Western blotting.

**Results:**

CEM/ara-G cells were 70-fold more ara-G-resistant than were CEM cells. CEM/ara-G cells were also refractory to ara-G-mediated apoptosis. The transcript level of human Equilibrative Nucleoside Transporter 1 was lowered, and the protein levels of deoxycytidine kinase and deoxyguanosine kinase were decreased in CEM/ara-G cells. The subsequent production of intracellular ara-GTP (21.3 pmol/10^7^ cells) was one-fourth that of CEM cells (83.9 pmol/10^7^ cells) after incubation for 6 h with 10 μM ara-G. Upon ara-G treatment, ara-G incorporation into nuclear and mitochondrial DNA resulted in the inhibition of DNA synthesis of both fractions in CEM cells. However, DNA synthesis was not inhibited in CEM/ara-G cells due to reduced ara-G incorporation into DNA. Mitochondrial DNA-depleted CEM cells, which were generated by treating CEM cells with ethidium bromide, were as sensitive to ara-G as CEM cells. Anti-apoptotic Bcl-xL was increased and pro-apoptotic Bax and Bad were reduced in CEM/ara-G cells.

**Conclusions:**

An ara-G-resistant CEM variant was successfully established. The mechanisms of resistance included reduced drug incorporation into nuclear DNA and antiapoptosis.

## Background

Nucleoside analogs belong to one of the most clinically useful and frequently used classes of agents for the treatment of hematological malignancies [1–6]. Nelarabine, 2-amino-9-β-D-arabinofuranosyl-6-methoxy-9H-purine, is a relatively new anticancer agent that targets T-cell malignancies, including T-cell acute lymphoblastic leukemia and T-cell lymphoblastic lymphoma [4–6]. The Cancer and Leukemia Group B conducted a phase 2 study of nelarabine for adult patients with relapsed or refractory T-cell leukemia/lymphoma [7]. Treatment with nelarabine resulted in a 41% response rate and a 31% complete remission rate. Although this clinical outcome is promising, nelarabine therapy should be further optimized by an improved understanding of its mechanism of action and by overcoming drug resistance.

Upon intravenous administration, nelarabine is demethylated to the active compound 9-β-D-arabinofuranosylguanine (ara-G) by adenosine deaminase in the plasma [4, 8–11]. Ara-G is transported into leukemic cells mainly via nitrobenzylthioinosine-sensitive nucleoside membrane transporter human Equilibrative Nucleoside Transporter 1 (hENT1) [12]. Ara-G is then phosphorylated to ara-G monophosphate by cytoplasmic deoxycytidine kinase (dCK) and mitochondrial deoxyguanosine kinase (dGK) [9]. This phosphorylation is the rate-limiting step of the intracellular activation of nelarabine. Ara-G nucleotide is partly dephosphorylated by cytosolic 5′-nucleotidase II (cN-II). Ara-G monophosphate is then phosphorylated to ara-G diphosphate and eventually to ara-G triphosphate (ara-GTP). Ara-GTP is an intracellular active metabolite, which is subsequently incorporated into both nuclear and mitochondrial DNA, thereby terminating DNA elongation. Thus, incorporation of the drug into DNA is critical for its cytotoxicity [8–10].

Nelarabine resistance is a major obstacle to improving response rates, and overcoming this drug resistance would provide new strategies for optimal nelarabine administration. In the present study, we established a novel ara-G-resistant subclone of the human T-cell lymphoblastic leukemia cell line, CCRF-CEM. Factors involved in the intracellular activation of ara-G that might be closely related to ara-G resistance [8–12], including hENT1, dCK, dGK, cN-II, and drug incorporation into DNA, were extensively investigated. Because ara-G is phosphorylated by cytoplasmic dCK and mitochondrial dGK, the contribution of both nuclear and mitochondrial DNA damage was evaluated. Moreover, because the induction of apoptosis is the final output of mechanism of ara-G cytotoxicity, the levels of apoptosis-related proteins were determined.

## Methods

### Reagents

Ara-G was purchased from R.I. Chemicals (Orange, CA, USA) and dissolved in 100% dimethyl sulfoxide. Standard ara-GTP was provided by GlaxoSmithKline, Japan (Tokyo, Japan). [5-^3^H] ara-G (30 Ci/mmol) was purchased from Moravek Biochemicals, Inc (Brea, CA, USA). Nine-β-D-arabinofucanosyl-2-fluoroadenine (F-ara-A) and cytarabine (ara-C) were purchased from Sigma-Aldrich (St Louis, MO, USA).

### Cell culture and development of an ara-G-resistant subclone

Human T-cell lymphoblastic leukemia CCRF-CEM cells were cultured in RPMI1640 media supplemented with 10% fetal calf serum. An ara-G-resistant variant, CEM/ara-G, was established by serial incubation of the cells with ara-G, followed by limiting dilution for cloning. In brief, the parental CEM cells were maintained with escalating concentrations of ara-G. The initial concentration (0.2 μM) was one tenth the concentration required to inhibit 50% growth of CEM cells (IC_50_). The cultures were observed daily and allowed to grow. In subsequent passages, the concentration of ara-G was gradually increased. Passaging was repeated for 10 months. When the ara-G concentration in the culture media reached 20 μM, one cell line resistant to ara-G (CEM/ara-G) was cloned by the limiting dilution method [13].

### Drug treatment

Both CEM and CEM/ara-G cells (2 × 10^6^ cells/ml, 10 ml) were incubated at 37°C with various concentrations of radiolabeled or non-labeled ara-G for the time periods indicated. Cells were then washed twice with PBS and centrifuged (500 × g, 5 min, 4°C) to collect the cell pellet.

### Proliferation assay

Growth inhibition effects were determined by the sodium 3′-(1-[(phenylamino)-carbonyl-3,4-tetrazolium])-bis(4-methoxy-6-nitro) benzene sulfonic acid hydrate (XTT) assay according to the manufacturer’s instructions (Roche, Indianapolis, IN, USA) with slight modifications [13].

Alternatively, the number of viable cells were quantitated as of the ATP present, which signals the presence of metabolically active cells, by using The CellTiter-Glo^®^ Luminescent Cell Viability Assay kit (Promega Corp., Madison, WI, USA). Briefly, the cell suspension having been treated were added to the reagent (1:1, v/v). The sample was mixed for 2 min for cell lysis, and allowed to stand for 10 min to stabilize the luminescent signal. The luminescence intensity of the sample was measured thereafter. This method was applied to assess the viability of mitochondrial DNA-depleted ρ^0^CEM cells.

### Measurement of analog triphosphate concentrations in leukemic cells

Intracellular concentrations of ara-GTP, F-ara-A triphosphate (F-ara-ATP), and ara-C triphosphate (ara-CTP) were determined by using the HPLC assay method that we previously established [13, 14]. Briefly, cells (1 × 10^6^ cells/ml, 10 ml) were incubated for 6 h with 10 μM ara-G, F-ara-A, or ara-C. The acid-soluble fraction, the nucleotide pool, was extracted from the cells by the addition of perchloric acid followed by neutralization. An aliquot of the sample was subjected to HPLC analysis. Chromatography was performed with the TSK gel DEAE-2 SW column (250 mm length × 4.6 mm inside diameter; Tosoh, Tokyo, Japan) and 0.06 M Na_2_HPO_4_ (pH 6.9) - 20% acetonitrile buffer at a constant flow rate of 0.7 ml/min. Each analog triphosphate concentration was quantitated by its peak area and expressed as pmol/10^7^ cells.

### Western blot analysis

Protein levels of dCK, dGK, caspase-3, caspase-9, Bcl2, Bcl-xL, Bax, Bad, Bid, Bim, AKT, and p-AKT were determined by using standard western blotting techniques [13]. Mouse monoclonal anti-dCK was developed in the Department of Pediatrics of Mie University School of Medicine [13]. Rabbit polyclonal anti-dGK antibody (Abgent, San Diego, CA, USA), rabbit polyclonal anti-caspase-3 (Cell Signaling Technology, Beverly, MA, USA), rabbit polyclonal anti-caspase-9 (Cell Signaling Technology), rabbit polyclonal anti-Bcl-2 (Cell Signaling Technology), rabbit polyclonal anti-Bcl-xL (Cell Signaling Technology), rabbit polyclonal anti-Bax (Cell Signaling Technology), rabbit polyclonal anti-Bad (Cell Signaling Technology), rabbit polyclonal anti-Bid (Cell Signaling Technology), rabbit polyclonal anti-Bim (Cell Signaling Technology), rabbit polyclonal anti-AKT (Cell Signaling Technology), rabbit polyclonal anti-P-AKT (Santa Cruz Biotechnology, Inc. Dallas, TX, USA), and anti-actin antibodies (Sigma-Aldrich) were used as primary antibodies [13].

### Determination of hENT1 and cN-II transcripts

To evaluate mRNA levels of hENT1 (accession: NM_001078177) and cN-II (accession: NM_012229), real-time RT-PCR was performed by using the ABI Prism 7900 sequence detection system (Applied Biosystems, Foster City, CA, USA) as previously described [13, 15]. Primers for hENT1 and cN-II were purchased from Applied Biosystems. The relative quantification method was used. The expression level of hENT1 or cN-II was normalized using β-Actin as a house-keeping gene in each cell line. The final value was expressed as the ratio of the expression level of hENT1 or cN-II of CEM/ara-G cells to that of CEM cells (the expression level of hENT1 or cN-II of CEM cells was set as 1).

### Calculation of ara-G incorporation into both nuclear and mitochondrial DNA

Both nuclear and mitochondrial DNA fractions were isolated from cells after incubation with tritiated ara-G for the indicated time periods at 37°C. For nuclear DNA isolation, the acid-insoluble fraction (obtained as described above) was used. To solubilize RNA, the acid-insoluble fraction was resuspended in 100 μl of 0.4 N KOH and kept at room temperature for 4 h. The sample was then mixed with 100 μl of 5% perchloric acid and 20 μl of 4 N HCl, followed by centrifugation (15,000 × g, 30 sec, 4°C). After removal of the supernatant (RNA), the precipitate was mixed with 100 μl of 5% perchloric acid and heated at 92°C for 20 min to solubilize DNA. After centrifugation (15,000 × g, 30 sec, 4°C), the supernatant was isolated as DNA, and the precipitate (protein) was discarded [16]. The mitochondrial fraction was extracted by using the Qproteome Mitochondria Isolation Kit (Qiagen, Valencia, CA, USA) according to the manufacturer’s instructions. Radioactivity was determined in both fractions by using a liquid scintillation counter.

### Evaluation of nuclear and mitochondrial DNA synthesis

The inhibition of DNA synthesis by ara-G was evaluated by assessing the incorporation of tritiated thymidine into DNA [17]. Cells (2 × 10^6^ cells) were pre-incubated with or without 10 μM ara-G for 3 h, followed by washing in fresh media and subsequent incubation with tritiated thymidine for 4 h. The nuclear and mitochondrial DNA fractions were extracted as described above and evaluated for radioactivity by using a liquid scintillation counter.

### Quantitation of apoptotic cell death

To evaluate cytotoxicity, apoptotic cell death was determined by staining for phosphatidylserine externalization by using annexin V (Roche Applied Science, Indianapolis, IN, USA) or for the sub-G1 cell cycle population by using propidium iodide (Beckman Coulter, Fullerton, CA, USA) and performing flow cytometry 72 h after treatment [18]. To confirm the induction of mitochondrial apoptosis, the cleavage of caspase-3 and caspase-9 was detected by western blotting as described above.

### Derivation of mitochondrial DNA-depleted cells (ρ^0^CEM cells)

CEM cells were cultured in the presence of 100 ng/ml ethidium bromide to inhibit mitochondrial DNA replication for more than 20 generations (almost 1 month) [19]. ρ^0^ cells were derived and maintained in the presence of 50 mg/ml uridine. The total cellular enzyme activity of cytochrome c oxidase, subunits of which are encoded by mitochondrial DNA, was tested by using the Mitochondrial Activity Assay Kit (BioChain, Institute, Inc., Hayward, CA, USA) according to the manufacturer’s instructions.

### Statistical analyses

All statistical analyses were performed with Microsoft Excel 2007 (Microsoft Corporation, Redmond, WA, USA). All graphs were generated using GraphPad Prism (version 5.0; GraphPad Software, San Diego, CA, USA).

## Results

### Establishment of ara-G-resistant CEM (CEM/ara-G) cells

**Figure 1:**
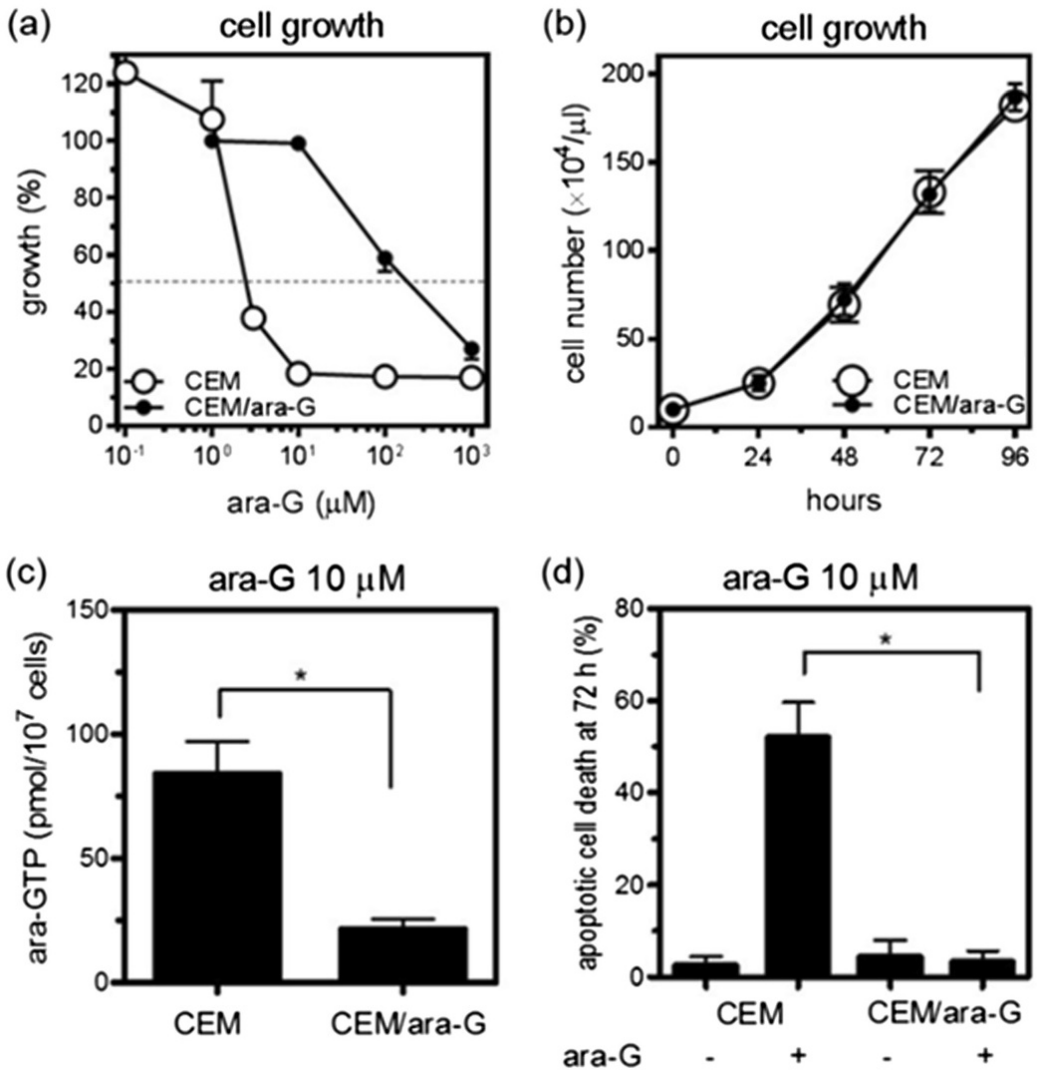
**Establishment of ara-G-resistant CEM variant, CEM/ara-G. (a)** The growth inhibition curve. Cells were incubated with various concentrations of ara-G for 72 h, and the IC_50_ was then determined by using the XTT assay. **(b)** Doubling time for CEM cells and CEM/ara-G cells. **(c)** Intracellular ara-GTP concentrations. CEM cells and CEM/ara-G cells were incubated for 6 h with 10 μM ara-G, followed by an extraction of the nucleotide pool and subsequent measurement of ara-GTP by using HPLC. *P = 0.0006 determined by unpaired *T* test. **(d)** Apoptotic cell death induced by ara-G. CEM cells and CEM/ara-G cells were incubated with 10 μM ara-G for 72 h, followed by the evaluation of annexin V positivity by flow cytometry. *P = 0.002 determined by unpaired *T* test. The values shown are the mean ± SD of at least three independent experiments.

**Figure 2:**
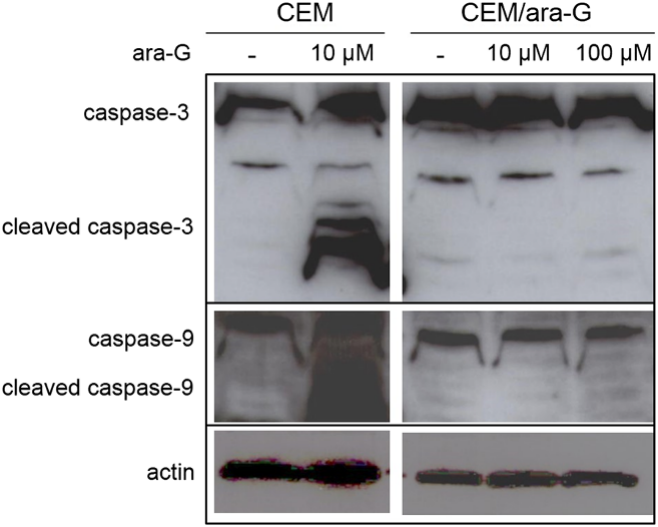
**Induction of apoptosis.** CEM cells and CEM/ara-G cells were incubated for 72 h with 10 or 100 μM ara-G, followed by the examination of caspase cleavage.

The XTT proliferation assay demonstrated that CEM/ara-G cells were 70-fold more resistant to ara-G than CEM cells (Figure 1a, Table 1). Because growth rates for both cell lines were similar (Figure 1b) with a doubling time of 22.0 h for CEM cells and 21.4 h for CEM/ara-G cells, the resistance to this S-phase-specific drug was not attributable to cycling speed. The intracellular ara-GTP production (21.3 pmol/10^7^ cells) was reduced by 1/4 in CEM/ara-G cells compared with that (83.9 pmol/10^7^ cells) in CEM cells (Figure 1c). CEM/ara-G cells were also resistant to ara-G-induced apoptosis (Figure 1d). Cleavage of caspase 3 and caspase 9 was demonstrated in CEM cells treated with ara-G, suggesting that mitochondria-mediated apoptosis was induced by ara-G (Figure 2). In contrast, caspase cleavage was not induced in CEM/ara-G cells treated with 100 μM ara-G (Figure 2). Thus, the ara-G-resistant CEM variant, CEM/ara-G, was successfully established, which yielded a small amount of ara-GTP and was consequently more resistant to ara-G-induced growth inhibition and apoptosis.

**Table 1:** **Drug sensitivities of CEM and CEM/ara-G cells**

Drug	IC _50_ (μM)		
	CEM	CEM/ara-G	RR
Ara-G	2.6	180	(70)
F-ara-A	0.10	4.80	(48)
Ara-C	0.15	0.75	(5)

CEM and CEM/ara-G cells were incubated for 72 h with various concentrations of ara-G, ara-C, or F-ara-A. The IC_50_ was then determined by using the XTT assay. The number in the parenthesis is the relative resistance (RR), which was obtained by dividing the IC_50_ value of CEM/ara-G cells by that of CEM cells.

### Cross-resistance in CEM/ara-G cells

**Figure 3:**
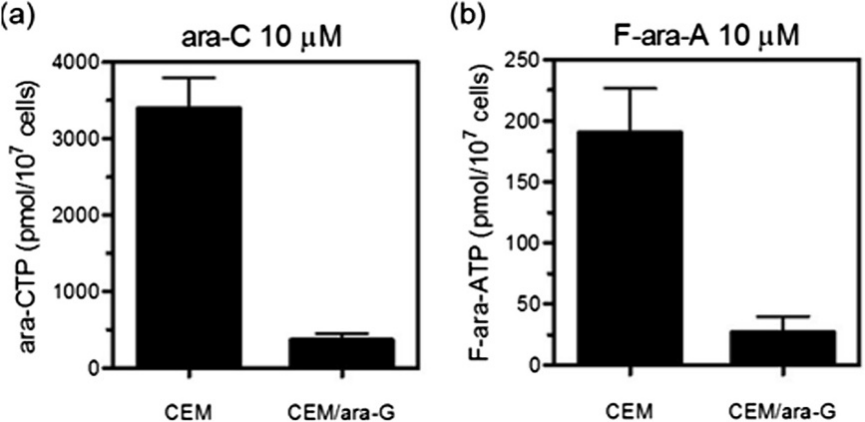
**Intracellular analog triphosphate production.** CEM cells and CEM/ara-G cells were incubated for 6 h with 10 μM ara-C **(a)** or F-ara-A **(b)**, followed by extraction of the nucleotide pool and measurement of intracellular analog triphosphate concentrations by using HPLC. P = 0.026 for CEM versus CEM/ara-G for ara-CTP production by unpaired *T* test **(a)**. P = 0.001 for CEM versus CEM/ara-G for F-ara-ATP production by unpaired *T* test **(b)**. The values shown are the means ± SD of at least three independent experiments.

The XTT assay also revealed that CEM/ara-G cells were cross-resistant to similar nucleoside analogs, ara-C and fludarabine nucleoside F-ara-A (Table 1). Intracellular analog triphosphate production was also determined. CEM/ara-G cells yielded lower amounts of both ara-CTP and F-ara-ATP than CEM cells (Figure 3). Ara-CTP and F-ara-ATP were 3,400 ± 400 pmol/10^7^ cells and 190 ± 36 pmol/10^7^ cells in CEM cells, and 363 ± 84 pmol/10^7^ cells and 29 ± 13 pmol/10^7^ cells in CEM/ara-G cells, respectively. Thus, the cross-resistance to ara-C and F-ara-A in CEM/ara-G cells was associated with the decreased production of intracellular analog triphosphates.

### Evaluation of factors (hENT1, dCK, dGK, and cN-II) essential for intracellular ara-GTP production

**Figure 4:**
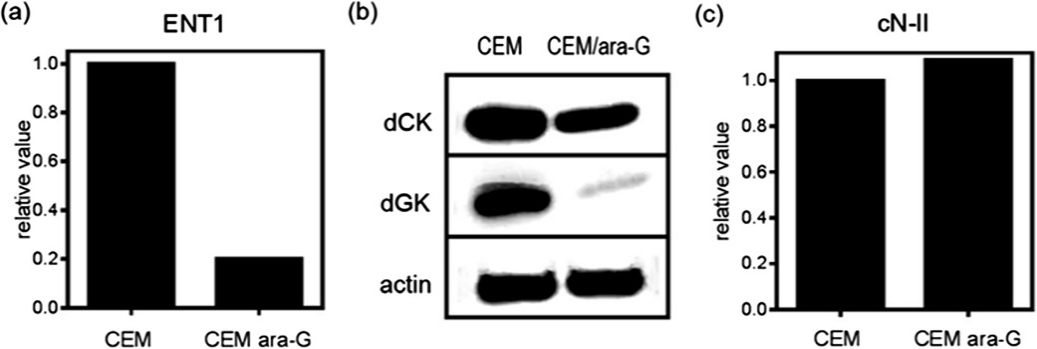
**Factors associated with the intracellular activation of ara-G in CEM cells and CEM/ara-G cells. (a)** Real-time RT-PCR was performed to determine the transcript level of hENT1. **(b)** Western blot analysis of dCK and dGK. **(c)** Real-time RT-PCR was performed to determine the transcript level of cN-II.

The mechanism of resistance to nucleoside analogs is usually associated with impaired production of intracellular analog triphosphate [20, 21]. The level of hENT1 transcript was decreased in CEM/ara-G cells (Figure 4a), suggesting a decreased cellular uptake of the nucleoside analog. Both dCK and dGK protein expression was also decreased in CEM/ara-G cells (Figure 4b). Transcript levels of the degrading enzyme cN-II were comparable between CEM cells and CEM/ara-G cells (Figure 4c). Thus, the cellular uptake and intracellular phosphorylation of ara-G were impaired in CEM/ara-G cells, which led to decreased ara-GTP production.

### Inhibition of DNA synthesis by the incorporation of ara-G into DNA

**Figure 5:**
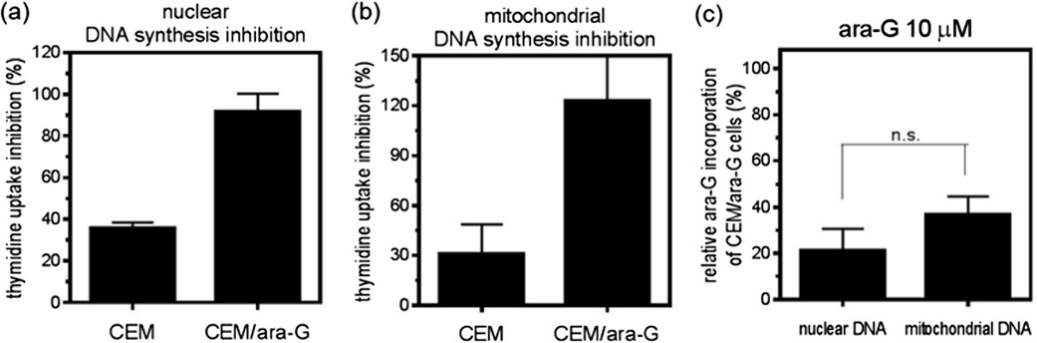
**DNA synthesis inhibition by ara-G.** CEM cells and CEM/ara-G cells were incubated with or without 10 μM ara-G for 3 h, followed by a 4-h incubation with tritiated thymidine. Nuclear **(a)** and mitochondrial **(b)** DNA fractions were isolated and subjected to scintillation counting. Percentages are the ratio of the values of thymidine incorporation into the DNA of the cells that had been pre-treated with ara-G relative to those without ara-G pre-incubation. P = 0.0003 for CEM versus CEM/ara-G for nuclear DNA synthesis inhibition by unpaired *T* test. P = 0.045 for CEM versus CEM/ara-G for mitochondrial DNA synthesis inhibition by unpaired *T* test. **(c)** CEM and CEM/ara-G cells were incubated with 10 μM radio-labeled ara-G for 6 h, followed by extraction of nuclear and mitochondrial DNA. Then, the samples were subjected to scintillation counting. The relative ara-G incorporation is the ratio of the value of ara-G incorporation into the DNA of CEM/ara-G cells to that of CEM cells. n.s., not significant. The values shown are the means ± SD of at least three independent experiments.

The critical cytotoxic event of a nucleoside analog is incorporation of the intracellular analog triphosphate into nuclear DNA, thereby terminating DNA synthesis [16, 22, 23]. The uptake of thymidine into DNA was evaluated in the presence or absence of ara-G in both cell lines. Pre-incubation with 10 μM ara-G, which is a concentration that is cytotoxic to CEM cells but not to CEM/ara-G cells, inhibited the incorporation of tritiated thymidine into both the nuclear and mitochondrial DNA fractions in CEM cells (Figure 5a, b). However, thymidine incorporation into DNA was not inhibited in either fraction of CEM/ara-G cells (Figure 5a, b). Along with DNA synthesis inhibition, ara-G incorporation into DNA was evaluated in the nuclear and mitochondrial fractions of both cell lines. After treatment with 10 μM ara-G, the amounts of ara-G incorporated into the DNA of both fractions of CEM/ara-G cells were reduced compared with those of CEM cells (Figure 5c). The reduction was comparable between the nuclear DNA and mitochondrial DNA fractions of CEM/ara-G cells (Figure 5c). The reduced incorporation of ara-G might correspond to the failed inhibition of thymidine incorporation (Figure 5a, b). Thus, CEM/ara-G cells were refractory to ara-G-mediated DNA synthesis inhibition of both nuclear and mitochondrial DNA fractions due to the reduced ara-G incorporation into DNA. The reduced ara-G incorporation might be attributable to the decreased production of intracellular ara-GTP in CEM/ara-G cells.

### Derivation of mitochondrial DNA-depleted cells (ρ^0^CEM cells)

**Figure 6:**
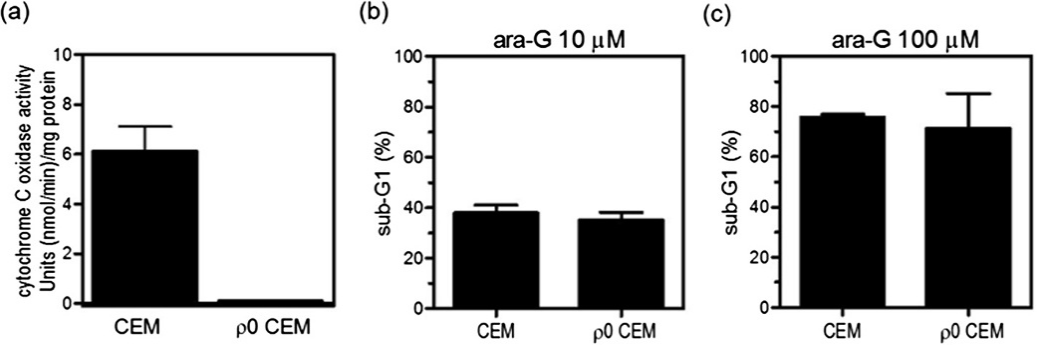
**Ara-G cytotoxicity against mitochondrial DNA-depleted CEM (ρ** ^**0**^ **CEM) cells. (a)** Determination of cytochrome c oxidase activity in ρ^0^CEM cells. The activity was completely suppressed in mitochondrial DNA-depleted variant cell line ρ^0^CEM as compared with CEM cells. **(b, c)** CEM cells and ρ^0^CEM cells were treated with 10 μM **(b)** or 100 μM **(c)** ara-G for 48 h. Sub-G1 induction was calculated by using flow cytometry. The values shown are the means ± SD of at least three independent experiments. The difference in the values between CEM cells and ρ^0^CEM cells was not significant for either concentration (P = 0.28 for 10 μM ara-G **(b)**, P = 0.40 for 100 μM ara-G **(c)**, unpaired *T* test).

The role of the mitochondrial DNA damage in ara-G cytotoxicity was further evaluated. If mitochondrial DNA is a target of ara-G cytotoxicity, it was hypothesized that mitochondrial DNA-depleted cells would become resistant to ara-G. CEM cells were cultured in the presence of ethidium bromide to generate a mitochondrial DNA-depleted derivative (ρ^0^CEM). The oxidase activity of cytochrome c, which is formed from subunits encoded by mitochondrial DNA, was almost absent in ρ^0^CEM cells (Figure 6a), indicating the successful depletion of mitochondrial DNA. The ATP-based proliferation assay revealed that the IC_50_ values were comparable between CEM cells and ρ^0^CEM cells (Table 2). The induction of apoptotic cell death was also evaluated in these cell lines. Intact mitochondrial function is not essential for inducing apoptosis because most ρ^0^cell lines undergo apoptosis in response to death signals and cytotoxic agents as efficiently as their parental cell lines [24–27]. Ara-G induced apoptosis equally in CEM cells and ρ^0^CEM cells, regardless of the ara-G concentration (Figure 6b, c). These results suggested that ara-G-induced mitochondrial DNA damage was unlikely to greatly contribute to ara-G cytotoxicity.

**Table 2:** **Drug sensitivity of CEM cells after the loss of mitochondrial DNA**

	IC _50_ (μM)	
Drug	CEM	ρ ^0^CEM
Ara-G	3.5	4.0

CEM cells and mitochondria-depleted ρ^**0**^CEM cells were incubated for 72 h with various concentrations of ara-G. The IC_50_ was then determined by using the ATP-based assay.

### Apoptosis-related proteins

**Figure 7:**
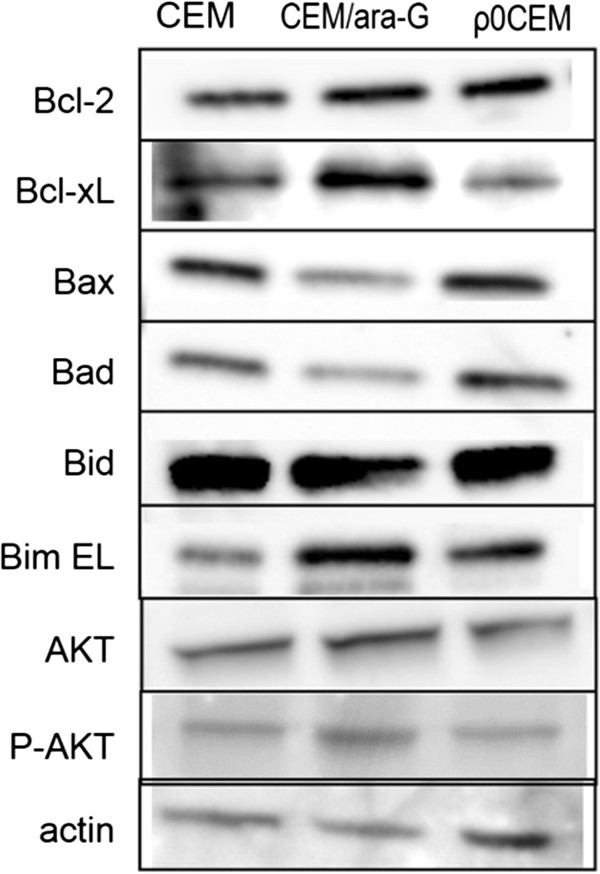
**Protein levels of Bcl-2, Bcl-xL, Bax, Bad, Bid, Bim EL (extra long), AKT, and phospho-AKT.** These levels were determined by Western blotting in CEM cells, CEM/ara-G cells, and ρ^0^CEM cells.

Apoptosis- and survival-related proteins were compared between CEM cells and CEM/ara-G cells (Figure 7). Anti-apoptotic Bcl-xL was augmented and pro-apoptotic Bax and Bad were reduced in CEM/ara-G cells, suggesting refractoriness to ara-G-induced apoptosis. The levels of mitochondrial apoptosis-related proteins, including Bcl-2, Bcl-xL, Bax, Bad, Bid, and Bim, were not altered in ρ^0^CEM cells. Pro-survival AKT and P-AKT levels were equivalent among CEM cells, CEM/ara-G cells, and ρ^0^CEM cells [28].

## Discussion

In the present study, we developed a new cell line variant of the T lymphoblastic leukemia CCRF-CEM cell line, which was resistant to ara-G, an active compound of nelarabine (Figures 1 and 2, Table 1), and investigated its mechanism of drug resistance. Reduced transporter hENT1 transcript level and decreased dCK and dGK protein levels (Figure 4) resulted in decreased ara-GTP production (Figure 1) in CEM/ara-G cells. The subsequent incorporation of ara-G into nuclear and mitochondrial DNA was reduced (Figure 5), and unable to inhibit DNA synthesis in both fractions of CEM/ara-G cells (Figure 5). Importantly, the cytotoxic effect of ara-G was almost unchanged on CEM cells that were depleted of mitochondrial DNA (Figure 6, Table 2), suggesting that mitochondrial DNA damage was unlikely to contribute greatly to ara-G cytotoxicity. Thus, the reduced triphosphate production (Figure 1) and the subsequent reduction of drug incorporation into nuclear DNA (Figure 5) were closely associated with the development of ara-G resistance in CEM/ara-G cells. The anti-apoptotic nature was also related to the drug resistance in this cell line (Figure 7).

Previously, 3 independent studies investigated the mechanisms of ara-G resistance in leukemic cell lines. Shewach et al. first developed an ara-G-resistant leukemic clone from T lymphoblastic leukemia MOLT-4 cells and demonstrated decreased production of intracellular ara-GTP [29]. However, they did not determine the mechanisms for the reduced ara-GTP production. Curbo et al. generated 2 ara-G-resistant CEM subclones that were 132-fold and 260-fold more ara-G resistant than CEM [30]. They demonstrated a decrease in ara-G incorporation into mitochondrial DNA and loss of dCK activity. However, they showed that the drug incorporation into mitochondrial DNA was not associated with the acute cytotoxicity induced by ara-G in their later study [31]. Their latest study further demonstrated that the depletion of mitochondria DNA does not attenuate the cytotoxicity of ara-G in MOLT-4 cells [32]. They concluded that the loss of dCK activity is the critical factor responsible for ara-G resistance. Our study demonstrated that ara-G inhibited both nuclear and mitochondrial DNA synthesis in CEM cells (Figure 5). However, the result showing that ρ^0^CEM cells were similarly sensitive to ara-G (Figure 6) suggests that the critical event should be the inhibition of nuclear DNA synthesis not mitochondrial DNA damage. Lotfi et al. developed 2 ara-G-resistant MOLT-4 variants that were 108-fold and 184-fold more ara-G resistant than MOLT-4 [33]. They showed that dGK deficiency was the most prominent change in these cells and that a dCK defect was associated with increased ara-G resistance [33]. They further identified increases in Bcl-xL in these ara-G-resistant clones [34]. The alteration of the kinases and anti-apoptotic Bcl-xL indicate a possible contribution of these factors to ara-G resistance, which is consistent with our present findings. Nevertheless, apart from these reports, we clearly showed all of the successive changes in the transporter hENT1, kinases (dCK and dGK), ara-GTP production, ara-G incorporation into nuclear and mitochondrial DNA, inhibition of DNA synthesis, and induction of mitochondria-mediated apoptosis. Thus, unlike previous studies, the present study was comprehensive and systematic in investigating the mechanism of resistance to ara-G in leukemic cells.

CEM/ara-G cells demonstrated cross-resistance to F-ara-A and ara-C. However, the resistance to the purine analog F-ara-A was much greater than that to the pyrimidine analog ara-C (Table 1). Because F-ara-A and ara-C share an identical pathway for their intracellular activation, the difference in resistance might be due to a structural difference between the 2 agents, but this possibility was not investigated in detail here. Nevertheless, one strategy to overcome ara-G resistance may be a high-dose ara-C therapy that can achieve 50-fold higher plasma ara-C concentrations than regular-dose ara-C, which would surpass the level of cross-resistance to ara-C [35, 36].

## Conclusions

An ara-G-resistant CEM variant was successfully established. The mechanism of resistance included reduced drug incorporation into nuclear DNA and antiapoptosis.

## Abbreviations

ara-G: 9-β-D-arabinofuranosylguanine
ara-GTP: 9-β-D-arabinofuranosylguanine triphosphate
F-ara-A: 9-β-D-arabinofucanosyl-2-fluoroadenine
F-ara-ATP: 9-β-D-arabinofucanosyl-2-fluoroadenine triphosphate
ara-C: Cytarabine
ara-CTP: Cytarabine triphosphate
XTT: Sodium 3′-(1-[(phenylamino)-carbonyl-3,4-tetrazolium])-bis(4-methoxy-6-nitro)benzene sulfonic acid hydrate
hENT1: Human Equilibrative Nucleoside Transporter 1
dCK: Deoxycytidine kinase
dGK: Deoxyguanosine kinase
cN-II: Cytosolic 5′-nucleotidase II
IC_50_: 50% growth-inhibitory concentration.
